# Risankizumab for Plaque and Guttate Psoriasis in a Patient with IgA-Related Glomerulonephritis

**DOI:** 10.5826/dpc.1104a100

**Published:** 2021-10-01

**Authors:** Annunziata Dattola, Arianna Zangrilli, Luca Bianchi

**Affiliations:** 1 Department of Dermatology University of Rome “Tor Vergata”, Rome, Italy

**Keywords:** guttate psoriasis, risankizumab, multi-failure, renal disease

## Introduction

Guttate psoriasis is a subtype of psoriasis characterized by acute eruption of numerous small, erythematous papules and plaques. It usually occurs in children and adolescents, but it can occur in other age groups. Streptococcus infection is an important risk factor which can usually precede its development by 2–3 weeks [1].

Risankizumab is a novel anti-interleukin 23 humanized antibody developed for severe plaque psoriasis. It is a humanized immunoglobulin monoclonal antibody (Ig) G1 selectively binding to and inhibiting p19 subunit of IL-23. No clinical cases are reported in literature regarding the use of risankizumab for guttate psoriasis’ treatment, but there is evidence reporting resolution of guttate psoriasis after 1 guselkumab injection, another novel anti-IL-23 [2].

Phase 3 trials evaluating the effectiveness and safety of risankizumab in patients with moderate-to-severe plaque psoriasis were: UltIMMa-1 (n = 506), UltIMMa-2 (n = 491), IMMhance (n = 507), and IMMvent (n = 605). In UltIMMa-1 and UltIMMa-2, randomized, double-blind, placebo-controlled, and active comparator-controlled trials, patients were randomized for risankizumab 150 mg, ustekinumab (45 mg or 90 mg), or placebo. Results showed a higher efficacy of risankizumab compared to ustekinumab and placebo; at week 16, psoriasis area and severity index 90 (PASI 90) was achieved by 75.3% and 74.8% of patients receiving risankizumab versus 42.0% and 47.5% receiving ustekinumab, respectively in UltIMMa-1 and UltIMMa-2 [3].

## Case Presentation

We describe a case of a 32-year-old female patient affected by plaque psoriasis at the level of the trunk and the extremities, covering approximately 30% of the patient’s body surface area (PASI score 18). She is also affected by “early stages” of glomerulonephritis IGA-related for 10 years, now under control without treatment.

The patient was treated with several topical treatments, phototherapy, systemic corticosteroids, and methotrexate for 6 months with no clinical efficacy. Cyclosporine was administered for glomerulonephritis. In 2016, after screening for biological treatment, she started treatment with etanercept 50 mg subcutaneous injection every week.

After 24 weeks of treatment, following the loss of efficacy with etanercept, we began adalimumab 40 mg subcutaneous injection therapy.

While the patient was undergoing adalimumab treatment, we observed a good control of cutaneous psoriasis (PASI residual 3) for 12 months. After 12 months with adalimumab the patient developed psoriatic lesions at the limbs and trunk; we decided to continue the treatment evaluating the patient at the next visit. At follow-up we noticed an important flair of the skin lesions, psoriatic plaques on the arms, and guttate psoriasis on the trunk. Adalimumab treatment was therefore discontinued.

Laboratory test did not detect abnormalities, streptozyme test was performed because of the appearance of guttate psoriasis and result was negative. Treatment with secukinumab was excluded because of recurrent genital candidiasis medical history. Ixekizumab was also excluded due to eczematous episodes localized on the patient’s face.

**Figure 1:**
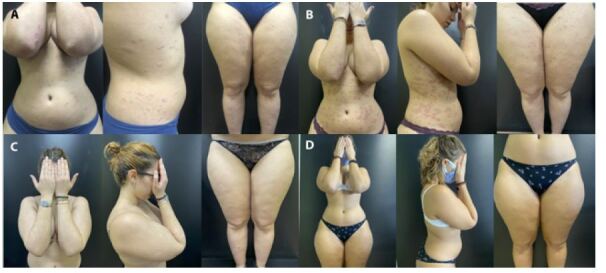
(A) Start of risankizumab treatment. (B) Clinical situation after 1 month of treatment following an episode of sore throat with positive streptozyme test. (C) Complete resolution of plaque psoriasis and guttate lesions after 16 weeks of treatment.

In January 2019 risankizumab therapy was started (150 mg subcutaneous injection) (Figure 1A). After 1 month of treatment followed by an episode of sore throat with positive streptozyme test treated only with *Streptococcus salivarius* probiotics, the patient’s clinical manifestations worsened, shifting from a PASI 12 to PASI 20 (Figure 1B). The patient was followed up to the next visit, and after 12 weeks of treatment, there was a reduction of PASI (PASI score 5) and good clinical improvement, with complete resolution of plaque psoriasis and guttate lesions after 16 weeks of treatment (PASI 0) (Figure 1C). Complete resolution of cutaneous plaque and guttate psoriasis was achieved after 12 months of treatment (Figure 1D).

## Conclusions

Psoriasis is a complex disease, caused by an inflammatory cascade involving cytokines as TNFα, and the IL-17/IL-23 axis. There are currently different treatment options to control the disease. According to new evidence, it is important to build personalized therapy considering the characteristics of psoriasis and the presence of co-morbidities that may affect the clinical response. Here we reported the effectiveness of risankizumab for both psoriatic plaque and guttate psoriasis.
